# The indirect impacts of nonpharmacological COVID-19 control measures on other infectious diseases in Yinchuan, Northwest China: a time series study

**DOI:** 10.1186/s12889-023-15878-3

**Published:** 2023-06-06

**Authors:** Weichen Liu, Ruonan Wang, Yan Li, Shi Zhao, Yaogeng Chen, Yu Zhao

**Affiliations:** 1 School of Public Health, Ningxia Medical University, Yinchuan, 750004 Ningxia China; 2 Key Laboratory of Environmental Factors and Chronic Disease Control, No. 1160, Shengli Street, Xingqing District, Yinchuan, 750004 Ningxia China; 3 School of Public Health, Chongqing Medical University, Chongqing, China; 4 Center for Disease Control and Prevention of Yinchuan, Yinchuan, 750004 Ningxia China; 5 JC School of Public Health and Primary Care, Chinese University of Hong Kong, Hong Kong, China; 6 CUHK Shenzhen Research Institute, Shenzhen, China; 7 School of Science, Ningxia Medical University, Yinchuan, 750004 Ningxia China

**Keywords:** COVID-19, Infectious diseases, Nonpharmaceutical intervention, China

## Abstract

**Background:**

Various nonpharmaceutical interventions (NPIs) against COVID-19 continue to have an impact on socioeconomic and population behaviour patterns. However, the effect of NPIs on notifiable infectious diseases remains inconclusive due to the variability of the disease spectrum, high-incidence endemic diseases and environmental factors across different geographical regions. Thus, it is of public health interest to explore the influence of NPIs on notifiable infectious diseases in Yinchuan, Northwest China.

**Methods:**

Based on data on notifiable infectious diseases (NIDs), air pollutants, meteorological data, and the number of health institutional personnel in Yinchuan, we first fitted dynamic regression time series models to the incidence of NIDs from 2013 to 2019 and then estimated the incidence for 2020. Then, we compared the projected time series data with the observed incidence of NIDs in 2020. We calculated the relative reduction in NIDs at different emergency response levels in 2020 to identify the impacts of NIPs on NIDs in Yinchuan.

**Results:**

A total of 15,711 cases of NIDs were reported in Yinchuan in 2020, which was 42.59% lower than the average annual number of cases from 2013 to 2019. Natural focal diseases and vector-borne infectious diseases showed an increasing trend, as the observed incidence in 2020 was 46.86% higher than the estimated cases. The observed number of cases changed in respiratory infectious diseases, intestinal infectious diseases and sexually transmitted or bloodborne diseases were 65.27%, 58.45% and 35.01% higher than the expected number, respectively. The NIDs with the highest reductions in each subgroup were hand, foot, and mouth disease (5854 cases), infectious diarrhoea (2157 cases) and scarlet fever (832 cases), respectively. In addition, it was also found that the expected relative reduction in NIDs in 2020 showed a decline across different emergency response levels, as the relative reduction dropped from 65.65% (95% CI: -65.86%, 80.84%) during the level 1 response to 52.72% (95% CI: 20.84%, 66.30%) during the level 3 response.

**Conclusions:**

The widespread implementation of NPIs in 2020 may have had significant inhibitory effects on the incidence of respiratory infectious diseases, intestinal infectious diseases and sexually transmitted or bloodborne diseases. The relative reduction in NIDs during different emergency response levels in 2020 showed a declining trend as the response level changed from level 1 to level 3. These results can serve as essential guidance for policy-makers and stakeholders to take specific actions to control infectious diseases and protect vulnerable populations in the future.

**Supplementary Information:**

The online version contains supplementary material available at 10.1186/s12889-023-15878-3.

## Introduction

2019 novel coronavirus disease (COVID-19) has had significant and devastating impacts on socioeconomic and human health worldwide [1–3]. As of March 6, 2022, over 433 million confirmed cases and over 5.9 million related deaths have been reported worldwide [4]. To address the ongoing COVID-19 pandemic, various nonpharmaceutical interventions (NPIs) measures have been implemented worldwide, including wearing medical masks, maintaining safe social distances, travel restrictions, crowd bans, quarantines, isolation, and increased personal precautions [5, 6]. Several studies have explored the impact of NPIs on airborne respiratory infectious diseases during the COVID-19 pandemic [7–13]; the recent decrease in other notifiable infectious diseases (NIDs) indicates the cobenefits of NPIs for other NIDs as well. For instance, in Germany [14] and Switzerland [15], with the exception of tick-borne encephalitis, the caseload of all other NIDs has declined. All infectious diseases in China demonstrated a downwards trend after the implementation of NPIs, with the incidences of respiratory, gastrointestinal and enteroviral diseases decreasing more steeply than the incidences of sexually transmitted, bloodborne, vector-borne and zoonotic diseases [5]. In the Taiwan region, the cases of NIDs have also decreased dramatically, with the exception of sexually transmitted diseases [2]. Xiao et al. [1] estimated that the caseload of 39 NIDs during the emergency response in 2020 was 65.6% lower than expected in Guangdong, with the greatest reductions observed for natural focal diseases and vector-borne infectious diseases. This evidence can provide valuable references to inform public health policy on the prevention and control of infectious diseases.

Ningxia Hui Autonomous Region (referred to as “Ningxia”) is located in northwest China and is characterized by a drought-semiarid climate. It is an economically underdeveloped region. Ningxia has become an exemplar in effectively implementing NPIs and was one of the provinces with the lowest incidence rate of COVID-19 in 2020. The daily number of newly confirmed cases of COVID-19 in Ningxia fluctuated at a low level after the first confirmed case was reported in Ningxia on January 22, 2020. As of March 3, 2020, no new confirmed case has been reported, and a total of 75 confirmed cases have been reported. Most studies have focused on areas with a high prevalence of COVID-19 [1, 2]; however, few studies have investigated low-incidence areas. In addition, the impacts of NPIs on notifiable infectious diseases during the COVID-19 pandemic were inconsistent due to the variability of the disease spectrum, high-incidence endemic diseases and environmental factors across different regions [5, 6, 9]. For instance, Ningxia has a high prevalence of brucellosis. Thus, it is of public health interest to explore the influence of NPIs on notifiable infectious diseases in Yinchuan, Northwest China.

Numerous studies have revealed that meteorological and pollution factors are associated with infectious diseases (vector-borne diseases, intestinal infectious diseases, respiratory infectious diseases, etc.) [16, 17]. For instance, the literature has reported that hand, foot and mouth disease is a climate-sensitive disease, and it is positively correlated with temperature, with some day lag [18, 19]. Climate affects the intensity of this disease by impacting the maintenance and replication of the pathogen, host, and vector populations; thus, temperature and precipitation have been shown to have effects in previous studies [20]. Ambient fine particles and temperature may affect the incidence and severity of respiratory infections by affecting vectors and host immune responses [21]. Most vector-borne diseases are sensitive to variations in meteorological factors, especially ambient temperature [22]. Meteorological and pollution factors are the strongest predictors of infectious diseases, including temperature, humidity, ambient fine particles, and ozone. In addition, infectious diseases are also influenced by the development of health infrastructure [23]. Thus, to improve the prediction accuracy of the incidence of infectious diseases in Ningxia, it is essential to incorporate meteorological and pollution factors and the development of health infrastructure into the prediction model of infectious diseases.

Therefore, in this paper, we aimed to assess the impacts of NPIs against the COVID-19 outbreak in 2020 on notifiable infectious diseases by using a dynamic regression model, which takes meteorological and pollution factors and health infrastructure into consideration. We analysed the effect of NPIs against COVID-19 on the changes in NID caseloads in Yinchuan under different geographic features. The results may complement the findings of NPIs in Northwest China and be essential for public health policy-makers and stakeholders to take specific actions to control infectious diseases and protect vulnerable populations in the future.

## Materials and methods

### Setting and data

This study selected the provincial capital of the Ningxia region, Yinchuan, as the study site. The data for 39 NIDs were obtained from Chinese Notifiable Infectious Disease Surveillance System (CNIDSS) during 2013-2020 and were classified into three categories of infectious diseases (Class A, Class B and Class C) according to the prevalence and risk level. All Class A NIDs and some Class B NIDs with a higher risk should be reported within 2 h, while other Class B NIDs and all C NIDs should be reported within 24 h [24]. The research further divided the NIDs into five groups based on different transmission routes [1]: respiratory infectious diseases; intestinal infectious diseases; sexually transmitted or bloodborne diseases; natural focal diseases and vector-borne infectious diseases and other infectious diseases (see Table S1). Due to the excessively low number of cases of other infectious diseases, we only analysed the top four categories of infectious diseases. Monthly average temperature, monthly average relative humidity, monthly average atmospheric pressure, and monthly average wind speed were collected from the National Meteorological Information Center (http://data.cma.gov). The monthly average pollutant concentrations gathered from the China Meteorological Science data sharing service system (http://hz.hjhj-e.com/home) during the same period included data on carbon monoxide (CO), nitrogen dioxide (NO_2_), ground-level ozone (O_3_), particulate matter less than or equal to 10 μm in aerodynamic diameter (PM_10_), particulate matter less than or equal to 2.5 μm in aerodynamic diameter (PM_2.5_) and sulfur dioxide (SO_2_). As the government continues to invest in health infrastructure, more health institutions and personnel can provide services for local residents. Thus, we selected the annual number of health institutional personnel in Yinchuan as a proxy variable to represent the development in health infrastructure (HIP). See Table S2 for details.

### NPIs

In 2020, in response to the COVID-19 pandemic, the Ningxia government initiated an emergency response to public health emergencies (from level 1 to level 3) according to the contingency plan for public health emergencies outlined in the Ningxia Hui Autonomous Region combined with the prevailing epidemic prevention and control situation [25, 26]. NPIs have been widely implemented worldwide as an important part of the public health response to outbreaks. The time frame of the emergency response levels from level 1 to level 3 for the COVID-19 outbreak in Ningxia [27, 28] is shown in Table S3.

### Statistical analysis

#### The time series model

The autoregressive integrated moving average (ARIMA) model is a time series prediction analysis method that is widely used to predict disease-related data due to its simplicity and practicality [29]. As mentioned above, the development of infectious diseases is influenced by meteorological, pollution factors and health infrastructure. To improve the accuracy of the predicted results, we combined multiple regression analysis with time series analysis to establish the ARIMA model with exogenous variables, namely, the dynamic regression (ARIMAX) model. The ARIMAX model considered infectious diseases as the response series and the indicators of pollutants and meteorological factors as the input series. Assuming that the response series $\{ {y_t}\}$ and the dependent variable series $\{ {x_{1t}}\} ,\{ {x_{2t}}\} ,?,\{ {x_{it}}\}$were smooth, the ARIMAX model was constructed as follows:

1 $$\left\{ \begin{gathered}  {y_t}=\mu +\sum\nolimits_{{i=1}}^{k} {\frac{{{\Theta _i}(B)}}{{{\Phi _i}(B)}}{B^{{l_i}}}{x_{it}}+{\varepsilon _t},}  \hfill \\  {\varepsilon _t}=\frac{{\Theta (B)}}{{\Phi (B)}}{\alpha _t}, \hfill \\ \end{gathered}  \right.$$

where ${y_t}$ is a response variable that denotes the monthly incidence of infectious diseases at time $t$, ${x_{it}}$ is an independent variable that denotes the corresponding *i*-th meteorological and pollution factor variables (temperature, relative humidity, atmospheric pressure, wind speed, CO, NO_2_, O_3_, PM_10_, PM_2.5_ and SO_2_) or HIP (the same annual data of HIP are used for different months of the same year). ${\varepsilon _t}$is the regression residual series,$\mu$ is an average term, ${\Phi _i}\left( B \right)$and$\Phi \left( B \right)$represent the autoregressive coefficient polynomials of the *i*-th individual variable and the residual series, respectively.${\Theta _i}\left( B \right)$and$\Theta \left( B \right)$are the moving average coefficient polynomials of the *i*-th individual variable and the residual series, respectively ${l_i}$ is the delayed order of the *i*-th individual variable and ${\alpha _t}$is a zero-mean white noise sequence. In addition, the Akaike information criterion (AIC) value and the mean absolute percentage error (MAPE) are used to determine the best model, which includes the combination of these factors, and to assess the prediction accuracy, respectively (Figure S2 and Figure S3).

#### The relative reduction of NIDs

We estimated the expected number of cases according to the optimal model (1) and then calculated the actual and expected relative reduction of NIDs with the following formula [1, 5]:

2 $$\begin{array}{l}{\rm{ARR}}{\mkern 1mu} ({\rm{Actual}}{\mkern 1mu} {\rm{relative}}{\mkern 1mu} {\rm{reduction}})(\% ) = \\\,\,\,\,\,\,\,\frac{{{N_{Avg}} - {N_{Real}}}}{{{N_{Avg}}}} \times 100\% ,\end{array} $$

3 $$\begin{array}{l}{\rm{ERR}}\,({\rm{Expected}}\,{\rm{relative}}\,{\rm{reduction}})(\% ) = \\\,\,\,\,\,\frac{{{N_{Pred}} - {N_{Real}}}}{{{N_{Pred}}}} \times 100\% \end{array} $$

where *N*_*Avg*_ and *N*_*Real*_ represent the average actual number of NIDs from 2013 to 2019 and the actual number of NIDs in 2020, respectively. *N*_*pred*_ represents the predicted number of NIDs during 2020.

A positive ARR or ERR indicates a reduction in NIDs under the effect of NIPs, while a negative value denotes an increase in NIDs under the effect of NPIs. The ARR is the indicator that the crude changes in NIDs are only depicted by comparison with historical data over the same period, and the ERR is the indicator that the changes in NIDs are adjusted by the ARIMAX model with environmental factors and HIP.

In this study, we first employed the ARIMAX model with environmental factors and HIP to predict the expected number of infectious diseases in 2020, and then the relative reduction in historical average years and expected NIDs compared to actual 2020 observations was examined. Next, we further compared the relative reduction in NIDs at different emergency response levels in 2020 to identify the cobenefits of NIPs on NIDs in Yinchuan. Then, we further selected the Baidu index by using the key word “mask” [30] as a proxy of the change in public awareness under the effect of NIPs (more details of the Baidu index and the reason why we selected the word “mask” are given in the Supplement). Taking respiratory infectious disease (influenza) as an example, we entered environmental factors, HIP and public awareness into the ARIMAX model to further explore the effect of NIPs on the relative change in NIDs.

#### Statistical software

The ARIMAX model was conducted using R (version 4.0.4) with the packages “*readxl*”, “*tsibble*”, “*TSA*”, “*tseries*”, “*fpp3*” and “*forecast*”. A two-sided P value less than 0.05 indicated statistical significance.

## Results

### The change in NIDs in Yinchuan between 2013 and 2020

**Fig. 1:**
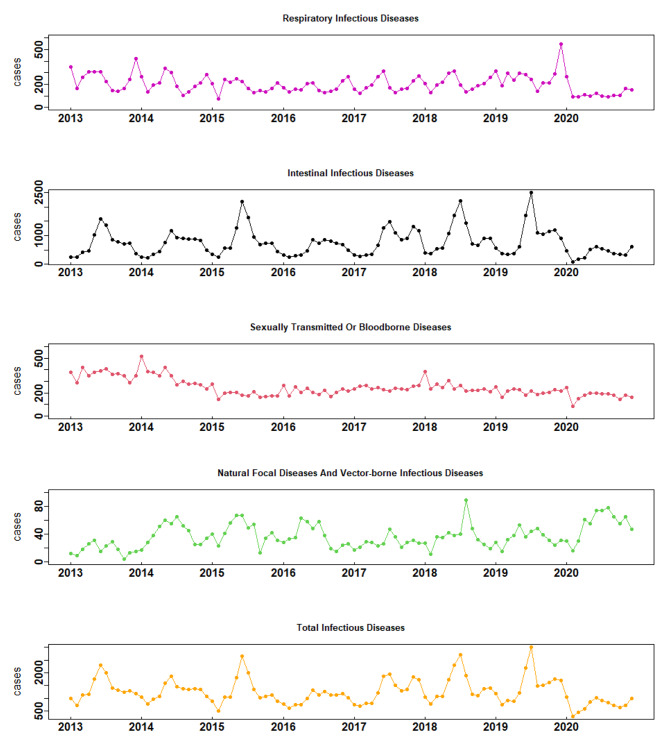
The incidence trend of five categories of notifiable infectious diseases in Yinchuan, China, from 2013 to 2020

Figure 1 depicts the time trends of five different types of NIDs in Yinchuan city from 2013 to 2020: respiratory infectious diseases, intestinal infectious diseases and natural focal diseases and vector-borne infectious diseases exhibited clear seasonality and showed an increasing trend in recent years. Sexually transmitted or bloodborne diseases presented a swinging decline until 2016 and then stabilized. The overarching trend of overall infectious diseases was consistent with that of intestinal infectious diseases, which accounted for the largest proportion of NIDs.

As shown in Table 1, a total of 9019 cases of NID were reported in Yinchuan in 2020, which was 42.59% lower than the average annual number of cases from 2013 to 2019. The largest reduction was observed for intestinal infectious diseases (50.62%), followed by respiratory infectious diseases (41.74%) and sexually transmitted or bloodborne diseases (31.74%), and there was an increase in natural focal diseases and vector-borne infectious diseases (-58.92%); the most common disease in each subgroup was hand, foot, and mouth disease (93.21%), scarlet fever (76.52%), hepatitis B (43.74%) and brucellosis (-77.36%), respectively.

**Table 1:** Comparison between the average cases of NIDs from 2013 to 2019 and the actual number in Yinchuan in 2020

Infectious disease	Before				Level 1				Level 2				Level 3				Total		
	2013–2019	2020	Relative Reduction (%)		2013–2019	2020	Relative Reduction (%)		2013–2019	2020	Relative Reduction (%)		2013–2019	2020	Relative Reduction (%)		2013–2019	2020	Relative Reduction (%)
**Overall**	**943**	**1026**	**-8.80**		**681**	**283**	**58.44**		**1898**	**1038**	**45.31**		**12,189**	**6672**	**45.26**		**15,711**	**9019**	**42.59**
**Respiratory infectious diseases**	**238**	**268**	**-12.61**		**136**	**91**	**33.09**		**436**	**201**	**53.90**		**1758**	**936**	**46.76**		**2568**	1496	**41.74**
Tuberculosis	74	56	24.32		54	43	20.37		150	129	14.00		448	391	12.72		726	619	14.74
Scarlet Fever	32	43	-34.38		13	3	76.92		80	10	87.50		599	114	80.97		724	170	76.52
Influenza	75	130	-73.33		28	19	32.14		37	14	62.16		167	72	56.89		307	235	23.45
Mumps	56	39	30.36		39	26	33.33		151	46	69.54		499	358	28.26		745	469	37.05
Others	1	0	-		2	0	100.00		18	2	88.89		45	1	97.78		66	3	95.45
**Intestinal infectious diseases**	**352**	**479**	**-36.08**		**288**	**89**	**69.10**		**841**	**417**	**50.42**		**8153**	**3772**	**53.73**		**9634**	**4757**	**50.62**
Dysentery	22	18	18.18		16	7	56.25		49	18	63.27		396	175	55.81		483	218	54.87
Hand-Foot-And-Mouth Disease	23	33	-43.48		10	1	90.00		129	10	92.25		3639	214	94.12		3801	258	93.21
Infectious Diarrhoea	305	427	-40.00		262	80	69.47		655	382	41.68		4088	3358	17.86		5310	4247	20.02
Others	2	1	-		0	1	-		8	7	12.50		30	25	16.67		40	34	15.00
**Sexually transmitted or bloodborne diseases**	**329**	**249**	**24.32**		**237**	**87**	**63.29**		**546**	**329**	**39.74**		**1988**	**1451**	**27.01**		**3100**	**2116**	**31.74**
HIV/AIDS	13	9	30.77		8	3	62.50		30	28	6.67		121	136	-12.40		172	176	-2.33
Gonorrhoea	13	14	-7.69		10	6	40.00		25	21	16.00		128	83	35.16		176	124	29.55
Syphilis	115	85	26.09		100	33	67.00		257	173	32.68		951	712	25.13		1423	1003	29.52
Hepatitis B	164	122	25.61		95	39	58.95		192	72	62.50		612	365	40.36		1063	598	43.74
Hepatitis C	24	19	20.83		24	6	75.00		42	35	16.67		176	155	11.93		266	215	19.17
**Natural focal diseases and** **vector-borne infectious diseases**	**24**	**30**	**-25.00**		**20**	**16**	**20.00**		**75**	**91**	**-21.33**		**290**	**513**	**-76.90**		**409**	**650**	**-58.92**
Brucellosis	19	25	-31.58		17	16	5.88		66	88	-33.33		247	490	-98.38		349	619	-77.36
Hydatid disease	5	4	20.00		3	0	100.00		8	1	87.50		22	16	27.27		38	21	44.74
Others	0	1	-		0	0	-		1	2	-		21	7	66.67		22	10	54.55

As shown in Table 2, the variations between the expected and actual numbers of natural focal diseases and vector-borne infectious diseases presented an increasing trend. The expected relative reduction in cases of natural focal and vector-borne infectious diseases in 2020 was approximately − 46.86% (95% CI: -523.74%, 16.77%), accompanied by 207 additional cases. On the other hand, the other types of NIDs exhibited declining patterns consistent with that of total infectious diseases, i.e., a relative reduction of 50.24% (95% CI: 8.61%,65.81%). Respiratory infectious diseases exhibited the strongest decline, as the actual incidence rate of 65.27% (95% CI: 47.36%, 74.08%) was lower than the expected incidence, which indicates a reduction of 2811 cases. There were also declines in intestinal infectious diseases (58.45%, 95% CI: -35.42%, 75.46%) and sexually transmitted or bloodborne diseases (35.01%, 95% CI: -23.21%, 55.86%), with reductions of 6692 cases and 1140 cases, respectively. The most common NIDs in these subgroups were hand, foot, and mouth disease (5854 cases), infectious diarrhoea (2157 cases) and scarlet fever (832 cases), respectively (more details in Table S4).

**Table 2:** The expected relative reduction (%) of observed NIDs compared to expected NIDs in Yinchuan in 2020

Infectious disease	Before (%) (95% CI)	Level 1 (%) (95% CI)	Level 2 (%) (95% CI)	Level 3 (%) (95% CI)	Total (%) (95% CI)
**Overall**	**16.32(-44.93,41.18)**	**65.65(-65.86,80.84)**	**45.95(-98.00,68.70)**	**52.72(20.84,66.30)**	**50.12(8.25,65.75)**
**Respiratory infectious diseases**	**38.8(23.87,48.83)**	**67.46(47.92,76.34)**	**73.14(61.71,79.31)**	**67.06(47.72,75.95)**	**65.27(47.36,74.08)**
Tuberculosis	18.66(-26.71,40.11)	38.79(5.69,54.69)	12.93(-30.5,34.67)	21.22(-30.71,43.62)	21.00(-26.91,42.65)
Scarlet Fever	28.36(-86.31,55.66)	90.85(-130.84,96.02)	92.18(74.31,95.38)	85.42(73.02,90.01)	83.04(64.19,88.89)
Influenza	40.02(8.84,55.30)	82.39(32.06,89.88)	87.76(-129.57,94.93)	76.80(-121.34,92.49)	68.64(-209.70,86.28)
Mumps	74.91(-746.66,41.92)	92.67(-607.78,66.75)	75.58(-25.54,76.13)	68.7(-349.01,54.64)	71.88(-276.25,58.40)
Others	100.00(-100.00,100.00)	100.00(-100.00,100.00)	-7.73(-105.05,95.38)	97.68(-100.81,99.52)	94.14(-101.56,98.98)
**Intestinal infectious diseases**	**-98.86(-324.73,31.07)**	**73.28(-127.52,91.01)**	**52.61(-187.61,81.35)**	**62.26(16.65,75.61)**	**58.45(-35.42,75.46)**
Dysentery	-25.21(-244.03,56.36)	34.47(-138.87,82.22)	36.54(-160.92,79.14)	40.75(-173.09,66.77)	37.5(-5311.22,68.57)
Hand-Foot-And-Mouth Disease	25.04(-109.20,92.62)	94.68(-100.20,99.81)	91.76(-101.08,99.15)	96.39(87.52,97.89)	95.78(-529.05,97.90)
Infectious Diarrhoea	13.52(-46.52,38.66)	80.32(51.69,87.65)	52.28(-20.09,70.22)	28.60(-21.11,49.37)	33.68(-19.71,54.13)
Others	-178.38(-116.14,72.55)	-360.89(-118.74,78.12)	-277.57(-183.08,42.31)	-178.89(-175.74,50.92)	-271.23(-164.20,52.30)
**Sexually transmitted or bloodborne diseases**	**4.80(-37.92,27.31)**	**65.38(45.05,74.73)**	**37.09(-5.22,55.13)**	**34.63(-36.14,56.99)**	**35.01(-23.21,55.86)**
HIV/AIDS	52.65(-11.18,69.92)	84.12(62.42,89.94)	25.81(-75.96,53)	10.61(-109.79,43.20)	22.74(-81.78,50.94)
Gonorrhoea	-145.97(-424.46,10.82)	2.88(-233.48,64.39)	-38.88(-366.98,44.89)	-38.5(-280.02,49.99)	-42.67(-297.51,47.59)
Syphilis	21.6(-20.53,41.90)	71.43(56.54,78.72)	26.2(-14.99,45.66)	17.5(-51.38,43.30)	24.09(-30.73,46.52)
Hepatitis B	-15.42(-134.26,23.42)	59.99(-6.77,75.38)	57.98(-136.75,76.94)	7.86(-199.81,68.48)	22.4(-342.43,66.55)
Hepatitis C	23.96(-43.4,48.26)	77.19(57.92,84.35)	34.53(-20.14,55.01)	24.51(-44.44,48.90)	30.66(-31.14,52.87)
**Natural focal diseases and** vector-borne **infectious diseases**	**-6.55(-910.87,43.76)**	**-21.06(-218.45,59.94)**	**-36.87(-768.49,25.71)**	**-53.25(-391.98,9.24)**	**-46.86(-523.74,16.77)**
Brucellosis	-6.69(-530.32,41.73)	14.39(-629.27,60.4)	-35.69(-468.22,22.96)	-63.92(-1181.11,12.44)	-52.5(-1032.07,18.24)
Hydatid disease	-89.09(-408.17,27.65)	100.00(-100.00,100.00)	81.91(-167.95,92.02)	-22.7(-205.82,61.16)	9.01(-211.08,67.73)
Others	55.52(-109.70,93.25)	100.00(-100.00,100.00)	75.44(-110.29,94.40)	81.76(-109.73,95.29)	80.5(-108.85,95.36)

# Before denotes the time frame before emergency response against COVID-19, Level 1, Level 2 and Level 3 are time frames corresponding to different emergency response levels. More details can be seen in Table S3

### The change in NIDs at different emergency response levels in 2020

**Fig. 2:**
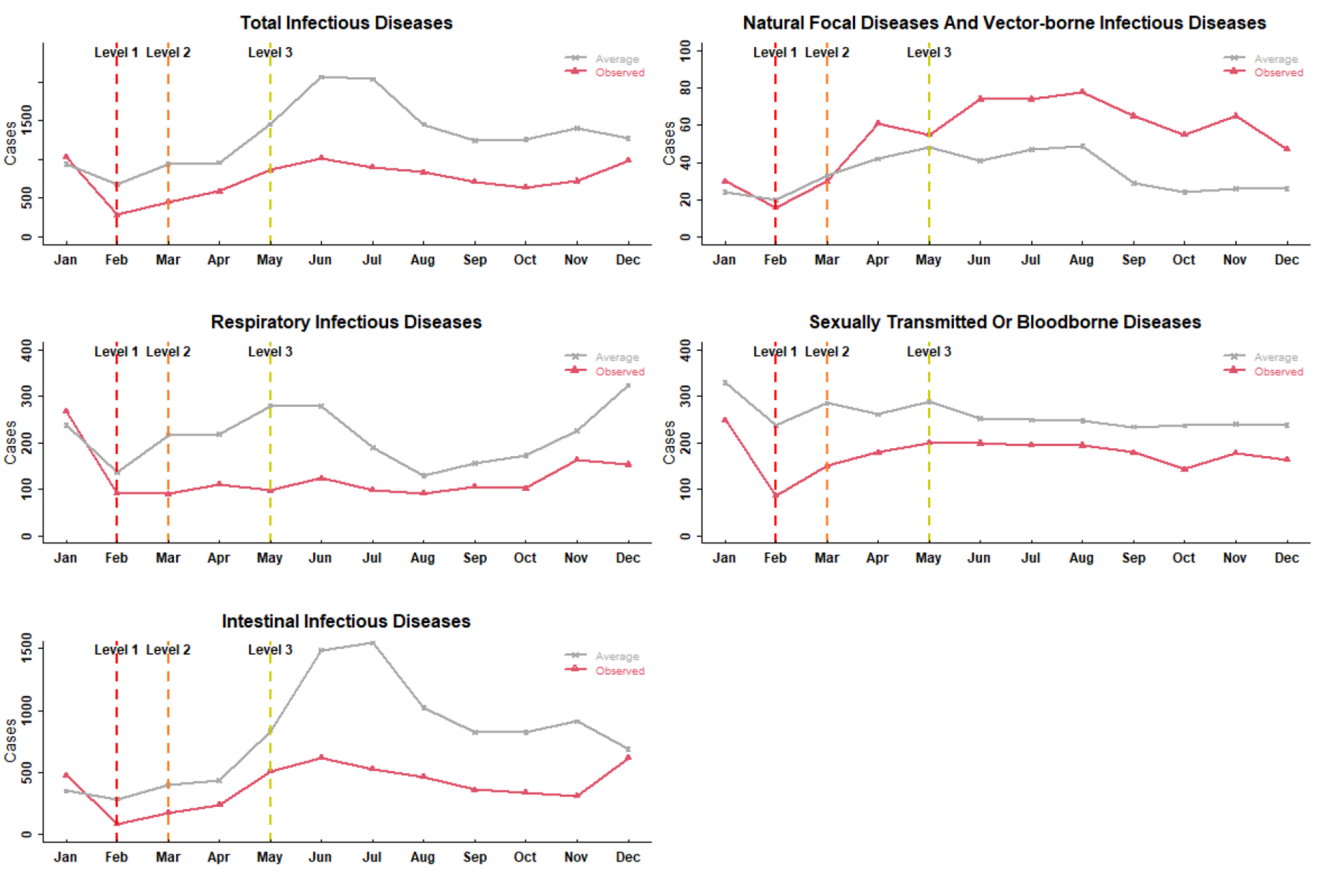
Comparison of the actual number of NIDs for different transmission routes in Yinchuan in 2020 with the synchronous period during 2015–2019

As shown in Fig. 2, with the activation of the emergency response, all the NIDs showed different degrees of decline but differed after the response of level 2, where the actual number of respiratory infectious diseases, intestinal infectious diseases and sexually transmitted diseases or bloodborne diseases were lower than the historical average from 2013 to 2019. The opposite trend was observed for natural focal diseases and vector-borne infectious diseases. This indicated that the change in the epidemiological characteristics of the above notifiable infectious diseases may be impacts by COVID-19. Thus, NPIs against COVID-19 may have inhibitory effects against respiratory infectious diseases, intestinal infectious diseases and sexually transmitted or bloodborne diseases.

**Fig. 3:**
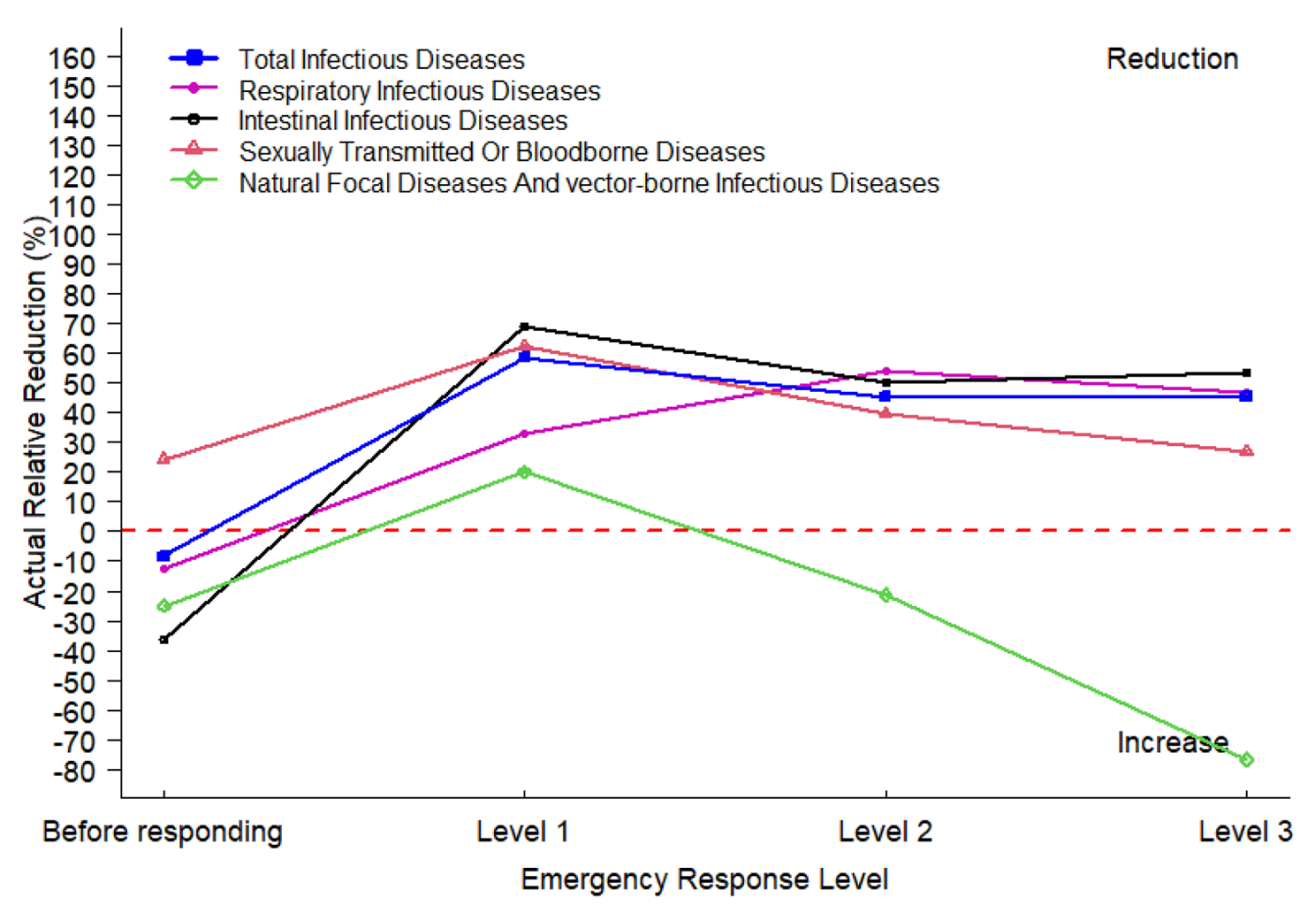
Actual relative reduction of notifiable infectious diseases through different transmission routes in Yinchuan during 2013–2020

The incidences of respiratory infectious diseases, intestinal infectious diseases and sexually transmitted or bloodborne diseases decreased in general during the whole period of emergency response, but the decreases varied across different emergency response levels. Respiratory infectious diseases had the largest decrease at level 2 (53.90%), followed by level 3 (46.76%) and level 1 (33.09%). Intestinal infectious diseases had the largest decrease at level 1, followed by level 3 and level 2. For sexually transmitted or bloodborne diseases, the rate of decrease fell as the level of emergency response decreased. Compared with the four categories of NIDs above, natural focal diseases and vector-borne infectious diseases showed the opposite trend, with reduction rates ranging from 20.00% (level 1) to -79.60% (level 3) (Table 1; Fig. 3).

**Fig. 4:**
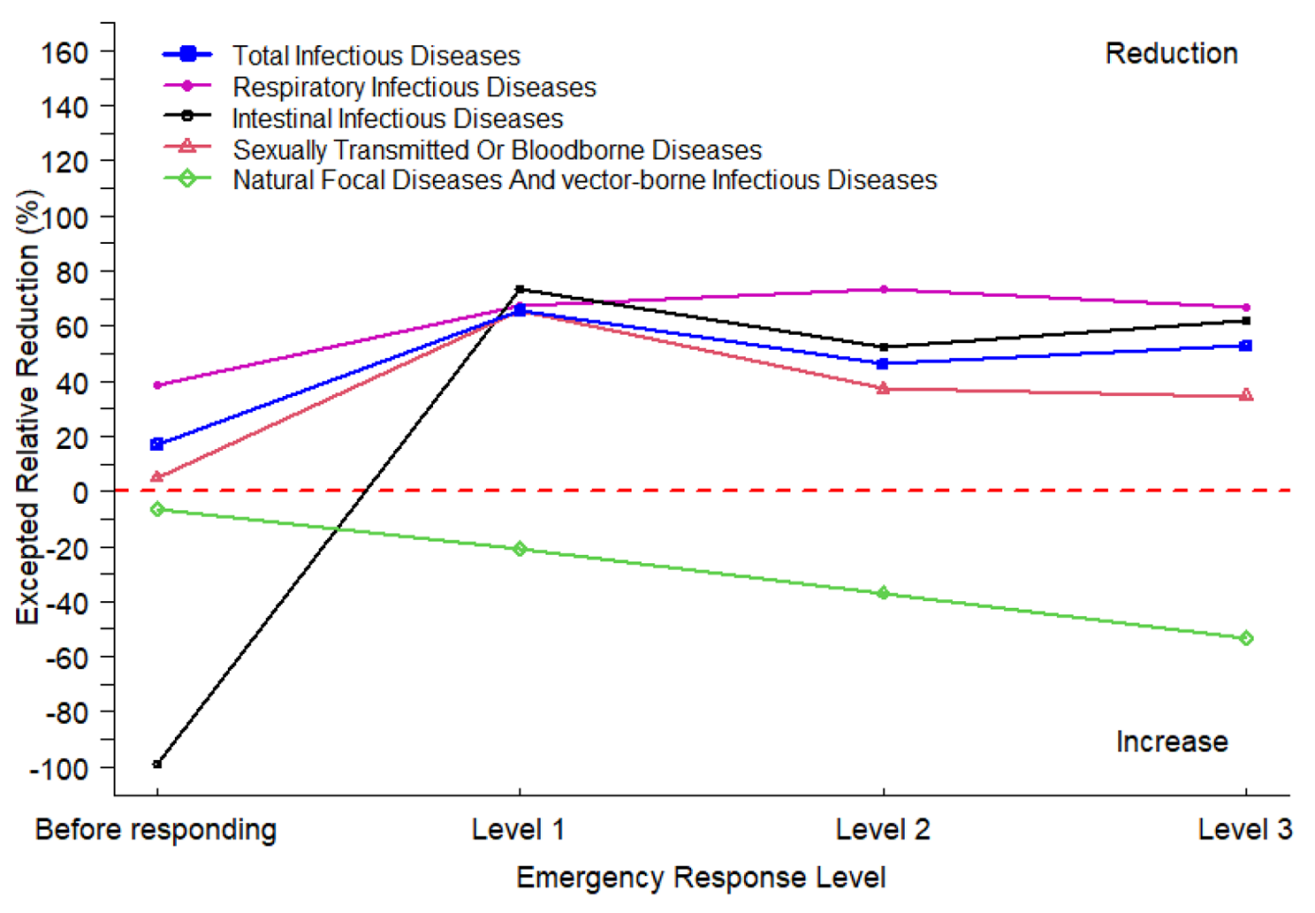
Expected relative reduction of notifiable infectious diseases through different transmission routes in Yinchuan at 2020

Table 2 shows the analysis of the reported and expected number of NIDs in Yinchuan in 2020. The findings indicate that the expected relative reduction in 2020 demonstrated a general downwards trend from 65.64% (95% CI: -65.61%, 80.83%) in the primary response to 52.82% (95% CI: 21.06%, 66.36%) in the tertiary response, with variations among the relative reduction in NIDs across different transmission routes. The greatest reductions in intestinal infectious diseases and sexually transmitted or bloodborne diseases were observed during the level 1 response (73.28% and 65.38%, respectively), while the greatest reduction in respiratory infectious diseases was observed during the level 2 response (73.14%). The relative reduction in respiratory infectious diseases, sexually transmitted or bloodborne diseases was lower in the level 3 response than in other response periods (67.06%, 34.63%), while the reduction in intestinal infectious diseases was lowest in the level 2 response period (52.61%). However, natural focal diseases and vector-borne infectious diseases gradually increased from level 1 (21.06%) to level 3 (53.25%) (as also shown in Fig. 4). Especially for brucellosis, we found that the NPIs only had an inhibitory effect on brucellosis in Yinchuan at level 1 (14.39%), and then the inhibitory effect disappeared at levels 2 (-35.69%) and 3 (-63.92%).

**Fig. 5:**
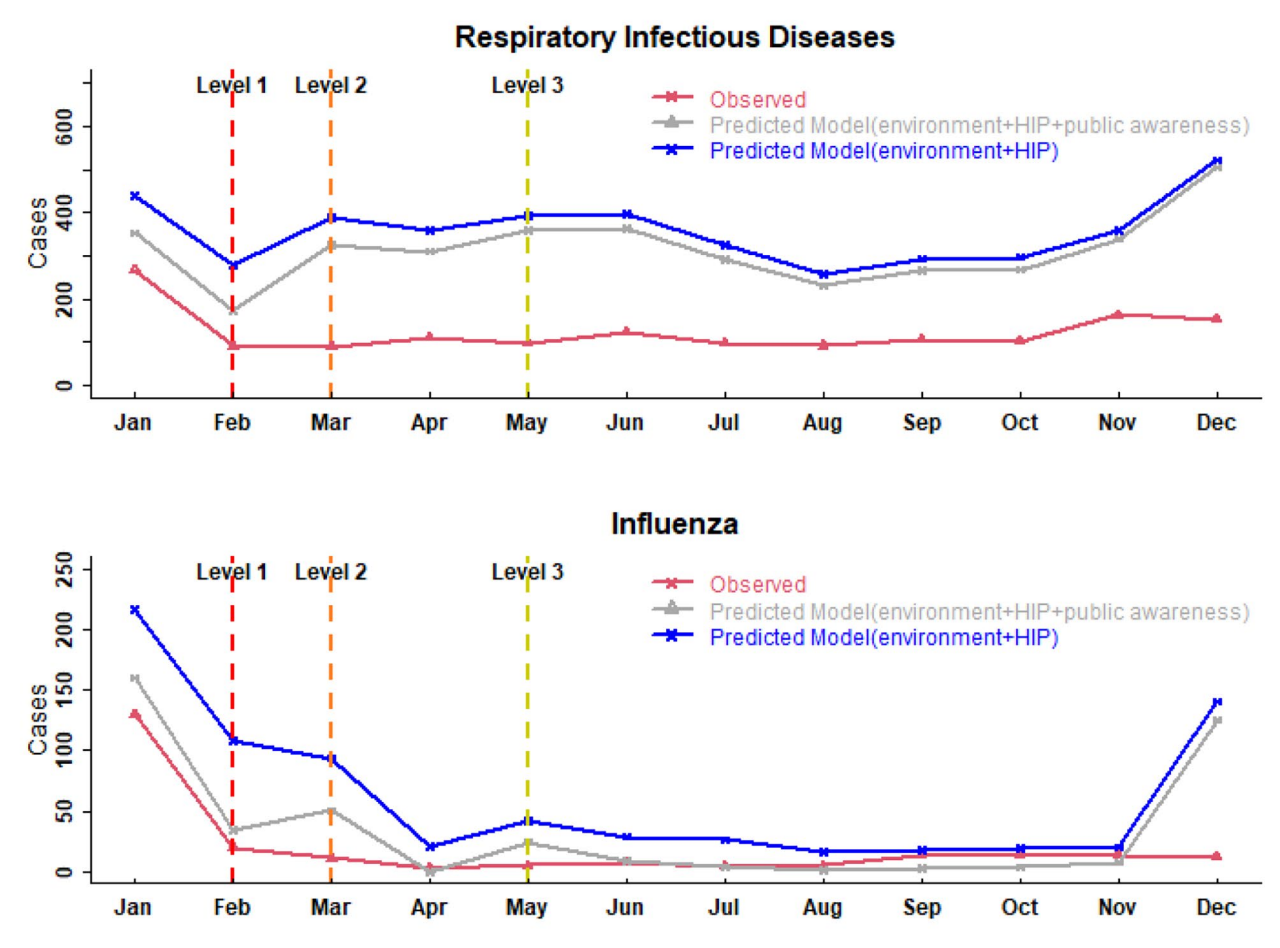
Comparison of the actual number of respiratory infectious diseases (influenza) with fitted values of different predicted models in Yinchuan in 2020. The red line represents the actual observed number, the gray line represents the predicted model with environmental factors, HIP and public awareness, the blue line represents the predicted model with environmental factors and HIP

Figure 5 shows a comparison of the actual number of respiratory infectious diseases (e.g., influenza) with fitted values of different predicted models in Yinchuan in 2020. We fitted two models to predict the number of total respiratory infectious diseases and influenza with different covariates (model 1 includes meteorological factors, pollution factors and HIP, and model 2 includes meteorological factors, pollution factors, HIP and public awareness). We observed that the fitted values of model 2 were closer to the actual number of total respiratory infectious diseases and influenza than those of model 1, which further demonstrated that the NPI-induced change in public awareness may be responsible for the reduction in respiratory infectious diseases.

## Discussion

The COVID-19 pandemic has continued to affect socioeconomic [31] and behavioural [32] patterns. To address the pandemic, several NPIs have been implemented in China, and positive results have been observed [33]. However, considering the large territory and population of China, the impacts of NPIs on NIDs are not completely consistent across regions, all of which have different policies as well as meteorological and pollution factors [5]. Therefore, it is worthwhile to explore the impacts of NPIs against COVID-19 implemented in different regions on the prevention and control of other notifiable infectious diseases. This study found that there was a notable reduction in NIDs observed during the COVID-19 pandemic compared to the historical average of NIDs and the predicted caseload of NIDs in 2020, which is consistent with several studies in China [1, 2, 5]. This reduction might be attributed to the positive response of the national public health policy in all regions of the country with various forms of NPIs, such as asking people to wear masks, enhancing health awareness of the population, limitations on gatherings, and strict travel restrictions.

With further analysis of the different transmission routes of NIDs, we found that NPIs for COVID-19 exhibited certain impacts on respiratory infectious diseases, intestinal infectious diseases and sexually transmitted or bloodborne diseases, but the degree of inhibition varied in strength. The impacts on the other NIDs were reflected by the significant reduction in cases, while the impacts on natural focal diseases and vector-borne infectious diseases were not significant. Additional relevant studies [1, 2, 5, 34] suggested that this difference in effects might be attributed to the fact that the degree of impact of NPIs on NIDs has different transmission patterns.

During the COVID-19 pandemic, several respiratory infectious diseases were significantly reduced, and they were mostly transmitted through interpersonal or airborne contact, which is consistent with the transmission route of COVID-19 [35]. Thus, the protective measures of wearing masks and maintaining social distance would suppress the spread of these respiratory diseases when combating COVID-19 [36]. We observed that scarlet fever, influenza, and mumps showed different levels of morbidity at different emergency response levels: a greater decrease at response level 2 than at response level 1 and a smaller decrease at response level 3. This indicated that the NPI in 2020 may have a delayed effect on controlling infectious disease [1]. Additionally, with the lower response levels and socioeconomic restart, the increased mobility of the population may result in a rebound for the general incidence levels, but the number of these diseases was still noticeably lower compared to previous years during the high incidence of respiratory diseases in winter, which is likely due to the postponed school openings [37] and wearing of masks [38], among other measures. One interesting finding was that the NIPs induced change of public awareness may be associated with the reduction of respiratory infectious diseases.

Similar to respiratory infectious diseases, intestinal infectious diseases also showed a dramatic decrease in caseload. During the periods of strict control, massive closures of schools as well as public places reduced the opportunities for person-to-person contact [36, 39], thereby reducing the risk of foodborne infections; additionally, changes in the personal hygiene habits of the population [40] further interrupted the spread of pathogen transmission. We also observed that the incidence of HFMD significantly decreased throughout 2020; the sharpest decline in this disease was observed in the third phase of the emergency response, when most diseases rebounded, which was different from previous studies. As a childhood susceptible disease, HFMD is easily transmitted among children under 5 years of age [41],and the delayed start of kindergarten [42, 43] coincided with missing the peak of the disease epidemic, which might be the reason for the low incidence of HFMD in this region in 2020.

Compared with respiratory infectious diseases or intestinal infectious diseases, the reduction in sexually transmitted or bloodborne diseases increased dramatically in response level 1 and then rebounded more rapidly. Several reasons could be attributed to this: patients interrupted the detection and treatment of diseases with worries about COVID-19 infection [44] and limitations of strict lockdown policies [45]; additionally, the reduction of high-risk sexual behaviour effectively reduced the growth of sexually transmitted diseases such as HIV and hepatitis B [46]. Nevertheless, the rebound of reduction in disease might be the result of progressive recovery of clinical services and sexual activity levels after the gradual control of the epidemic [47].

For natural focal diseases and vector-borne infectious diseases, although the level of exposure to these diseases among the population was sharply reduced, contact with infectious vectors or hosts is the major route of zoonotic disease transmission, and several NPIs (such as wearing masks and hand washing) implemented during the routine phase of the COVID-19 pandemic had limited effects on zoonotic disease prevention and control [48]. We observed significant increase in natural focal diseases and vector-borne infectious diseases (such as brucellosis) after response level 1. There are some reasons for this increase. First, the restrictions on human mobility in level 1 inevitably reduced people’s outdoor activities and hence lowered their exposure to vectors and animal hosts of vector-borne or zoonotic diseases [5]. Second, livestock production in Ningxia is mainly focused on sheep, and the virulence of Brucella in sheep is more potent [49]. Summer and spring are the peak periods of livestock breeding, and contact between the relevant occupational personnel and the abortive products and secretions of diseased animals dramatically enhance the onset of brucellosis [50].

However, this study still had some limitations. First, the congestion of medical resources by COVID-19 and the implementation of strict community control measures might result in underreporting of some notifiable infectious diseases. Second, the data were based on Yinchuan city for the study, and some of the NIDs were merged for analysis due to the low counts. Third, the modelling data were averaged by month, which probably resulted in some biases in the prediction. Finally, the results of this study cannot prove the causal association because of the ecological study design, and the outcomes should be further examined by more rigorous studies in the future (e.g., interrupted time series).

## Conclusion

The widespread implementation of NPIs in 2020 impacted the prevention and control of most NIDs, including relatively significant inhibitory effects on the incidence of respiratory infectious diseases, intestinal infectious diseases and sexually transmitted or bloodborne diseases, while the impact on natural focal diseases and vector-borne infectious diseases was limited. The relative reduction in NIDs during different emergency response levels in 2020 showed a declining trend as the response levels changed from level 1 to level 3. These results can serve as guidance for policy-makers and stakeholders to take specific actions to control infectious diseases and protect vulnerable populations in the future.

## Electronic supplementary material

Below is the link to the electronic supplementary material.

Supplementary Material 1

## Data Availability

The datasets used and/or analysed during the current study are available from the corresponding author upon reasonable request.
