# Supplementary material for: The indirect impacts of nonpharmacological COVID-19 control measures on other infectious diseases in Yinchuan, Northwest China: a time series study
Source: BMC Public Health. 2023 Jun 6;23:1089. doi: 10.1186/s12889-023-15878-3 (PMC10242608; doi:10.1186/s12889-023-15878-3)
Supplement: Supplementary file 1 — Supplementary Material 1 [file 12889_2023_15878_MOESM1_ESM.docx]

Appendix. Supplementary materials

**Table S1.** Name and classification of 39 types of NIDs in this study

| **No.** | **Notifiable infectious diseases** | **Group** |
| --- | --- | --- |
| **Respiratory infectious disease** | | |
| 1 | Human Infection with Avian Influenza H7N9 | Class B |
| 2 | Pertussis | Class B |
| 3 | Diphtheria | Class B |
| 4 | Tuberculosis | Class B |
| 5 | Meningococcal Meningitis | Class B |
| 6 | Scarlet Fever | Class B |
| 7 | atypical pneumonia | Class B |
| 8 | Measles | Class B |
| 9 | Influenza | Class C |
| 10 | Mumps | Class C |
| 11 | Rubella | Class C |
| 12 | Leprosy | Class C |
| **Intestinal infectious disease** | | |
| 13 | Cholera | Class A |
| 14 | Dysentery | Class B |
| 15 | Typhoid And Paratyphoid Fever | Class B |
| 16 | Poliomyelitis | Class B |
| 17 | Acute Hemorrhagic Conjunctivitis | Class C |
| 18 | Hand-Foot-And-Mouth Disease | Class C |
| 19 | Infectious Diarrhoea^*^ | Class C |
| **Sexually transmitted or blood-borne diseases** | | |
| 20 | HIV/AIDS | Class B |
| 21 | Gonorrhoea | Class B |
| 22 | Syphilis | Class B |
| 23 | Hepatitis B and Hepatitis C | Class B |
| **Natural focal diseases and vector-borne infectious diseases** | | |
| 24 | Plague | Class A |
| 25 | Anthrax | Class B |
| 26 | Schistosomiasis | Class B |
| 27 | Epidurals and fever | Class B |
| 28 | Leptospirosis | Class B |
| 29 | Malaria | Class B |
| 30 | Rabies | Class B |
| 31 | Brucellosis | Class B |
| 32 | Highly Pathogenic Avian Influenza | Class B |
| 33 | Encephalitis | Class B |
| 34 | Dengue Fever. | Class B |
| 35 | Typhus | Class C |
| 36 | Kala-azar | Class C |
| 37 | Hydatid disease | Class C |
| 38 | Filariasis | Class C |
| **Other infectious diseases** | | |
| 39 | Neonatal tetanus | Class B |

*Infectious diarrhoea here does not include cholera, dysentery, typhoid and paratyphoid fever.

**Table S2.** Descriptive analysis of environmental factors and health infrastructure in Yinchuan, 2013–2020.

| Variable | Mean (SD) | Min. | Median | Max. |
| --- | --- | --- | --- | --- |
| Temperature (℃) | 10.91(10.66) | -8.25 | 12.24 | 25.71 |
| Relative humidity (%) | 46.94(11.53) | 22.50 | 47.52 | 75.70 |
| Wind Speed (m/s) | 1.64(0.49) | 0.82 | 1.65 | 5.25 |
| Atmospheric pressure (hPa) | 890.52(5.13) | 881.10 | 891.00 | 899.00 |
| CO (mg/m3) | 0.95(0.33) | 0.50 | 0.83 | 2.01 |
| NO_2_ (µg/m3) | 33.96(9.74) | 18.52 | 31.68 | 57.26 |
| O_3_ (µg/m3) | 93.43(31.49) | 39.87 | 89.63 | 163.35 |
| SO_2_ (µg/m3) | 38.57(33.27) | 9.52 | 22.88 | 135.31 |
| PM_2.5_ (µg/m3) | 43.60(16.48) | 17.98 | 40.82 | 87.15 |
| PM_10_ (µg/m3) | 102.10(31.14) | 45.25 | 99.74 | 168.53 |
| HIP^#^(person) | 27973.25(4220.15) | 21621 | 27890 | 34234 |

**#** HIP: annual number of health institutional personnel.

**Table S3.** Delineation of emergency response levels for COVID-19 in Ningxia, 2020.

| Emergency response level | Time frame | | The time frame of the study |
| --- | --- | --- | --- |
|  | Start | End |  |
| Before | 0:00 on 1 January | 19:00 on January 25 | January |
| Level 1 | 19:00 on January 25 | 18:00 on February 28 | February |
| Level 2 | 18:00 on February 28 | 17:00 on May 6 | March to April |
| Level 3 | 17:00 on May 6 | 24:00 on December 31 | May to December |

**Table S4.** The difference between expected and observed counts for NIDs during 2020 in Yinchuan.

| **Infectious disease** | **2020** | **Expected cases (95%CI)** | **Difference (95%CI)*** |
| --- | --- | --- | --- |
| **Overall** | **9019** | **18109(9845,26373)** | **9090(826,17354)** |
| **Respiratory infectious disease** | **1496** | **4307(2842,5773)** | **2811(1346,4277)** |
| Tuberculosis | 619 | 784(488,1079) | 165(-131,460) |
| Scarlet Fever | 170 | 1002(475,1530) | 832(305,1360) |
| Influenza | 235 | 749(-214,1713) | 514(-449,1478) |
| Mumps | 469 | 626(125,1127) | 157(-344,658) |
| Others | 3 | 51(-193,295) | 48(-196,292) |
| **Intestinal infectious disease** | **4757** | **11449(3513,19386)** | **6692(-1244,14629)** |
| Dysentery | 218 | 349(4,694) | 131(-214,476) |
| Hand-Foot-And-Mouth Disease | 258 | 6112(-60,12283) | 5854(-318,12025) |
| Infectious Diarrhoea | 4247 | 6404(3548,9260) | 2157(-699,5013) |
| Others | 34 | 9(-53,71) | -25(-87,37) |
| **Sexually transmitted or bloodborne diseases** | **2116** | **3256(1717,4794)** | **1140(-399,2678)** |
| HIV/AIDS | 176 | 228(97,359) | 52(-79,183) |
| Gonorrhoea | 124 | 87(-63,237) | -37(-187,113) |
| Syphilis | 1003 | 1321(767,1875) | 318(-236,872) |
| **Hepatitis B** | 598 | 771(-247,1788) | 173(-845,1190) |
| **Hepatitis C** | 215 | 310(164,456) | 95(-51,241) |
| **Natural focal diseases and vector-borne infectious diseases** | **650** | **443(104,781)** | **-207(-546,131)** |
| Brucellosis | 619 | 406(55,757) | -213(-564,138) |
| Hydatid disease | 21 | 23(-19,65) | 2(-40,44) |
| Others | 10 | 51(-113,216) | 41(-123,206) |

*Difference= expected cases - observed cases, Difference>0 represent the decrease of NIDS, while Difference<0 represent the increase of NIDS.

**
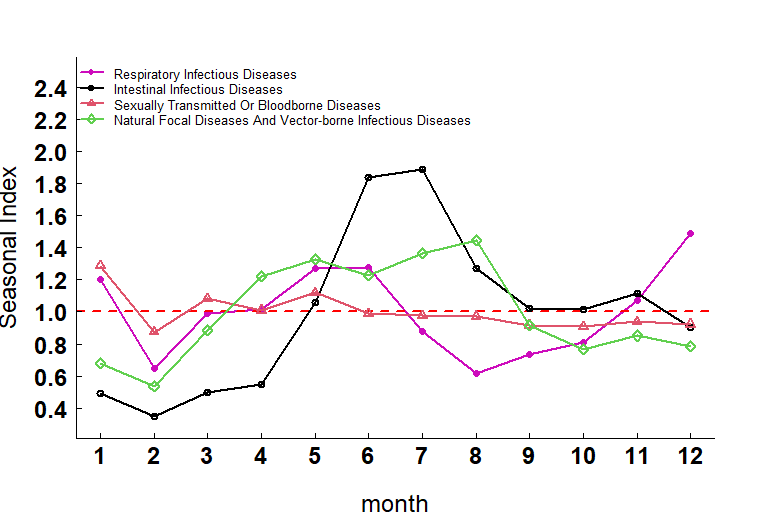
Figure S1** Seasonal index of the monthly numbers of four categories of notifiable infectious diseases in Yinchuan from 2013 to 2020

The monthly incidence numbers of NIDs in Yinchuan from 2013 to 2020 and seasonal indexes were shown in Figure S1. The seasonal trends of different transmission routes of NIDs were not the same: respiratory infectious diseases, intestinal infectious diseases, and natural focal diseases and vector-borne infectious diseases exhibited stronger seasonal trends. The risk of respiratory infectious diseases was higher in May, June and December, and lower in February and August, with December being roughly 2.4 times the risk of August. For intestinal infectious diseases, the highest incidence risk was 5.4 times higher in July than the lowest incidence risk in February. The risk of natural focal diseases and vector-borne infectious diseases was lowest in February, then progressively increased to peak in August and declined later, with the risk of incidence in August approximately 2.72 times higher than that in February. There was no significant seasonal trend for sexually transmitted or bloodborne diseases.

**Figure S2** The observed and fitted trends of notifiable infectious diseases stratified by disease classification from 2013 to 2020 in Yinchuan, China.


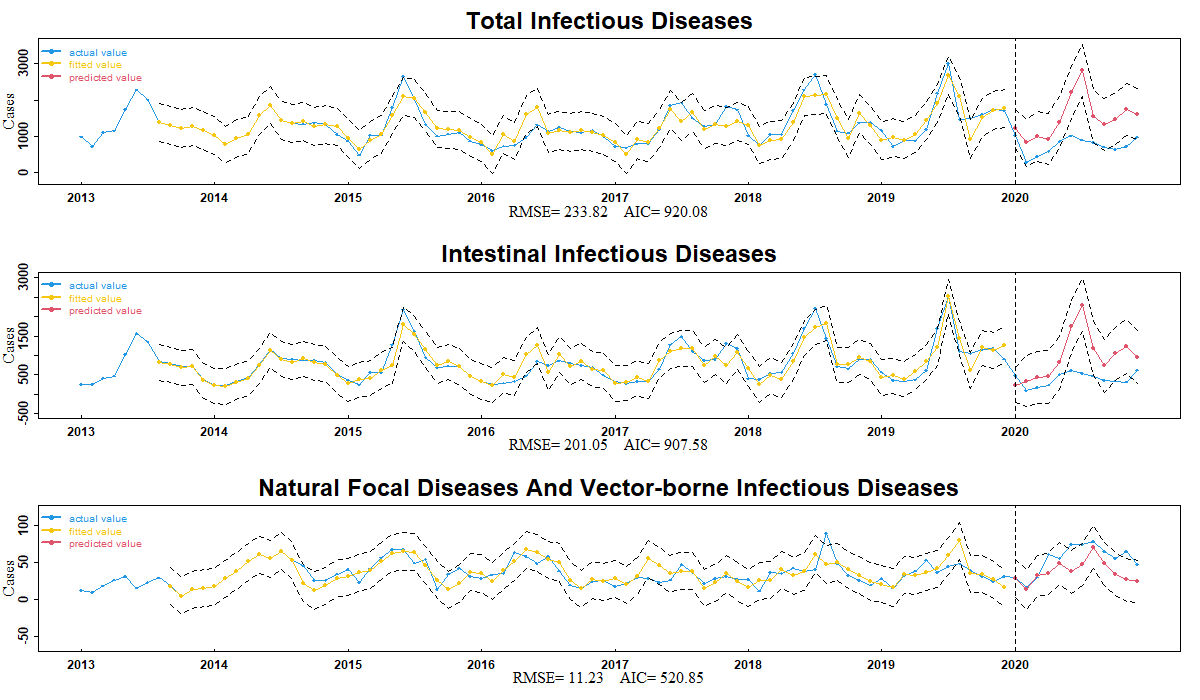

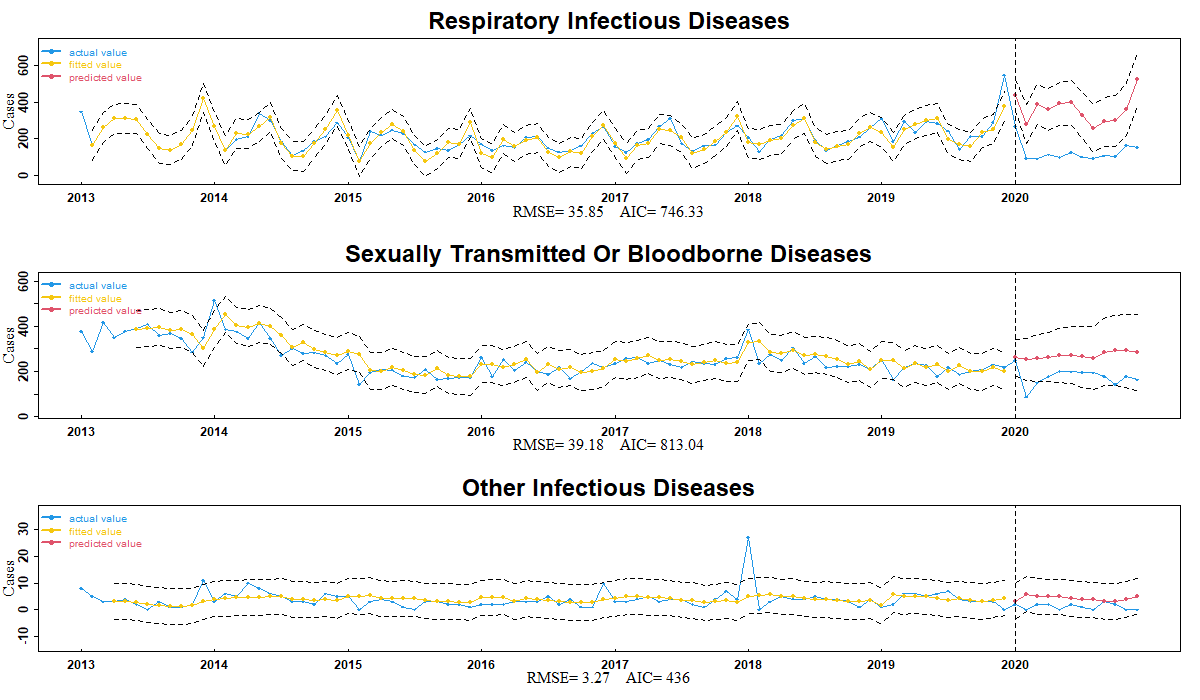


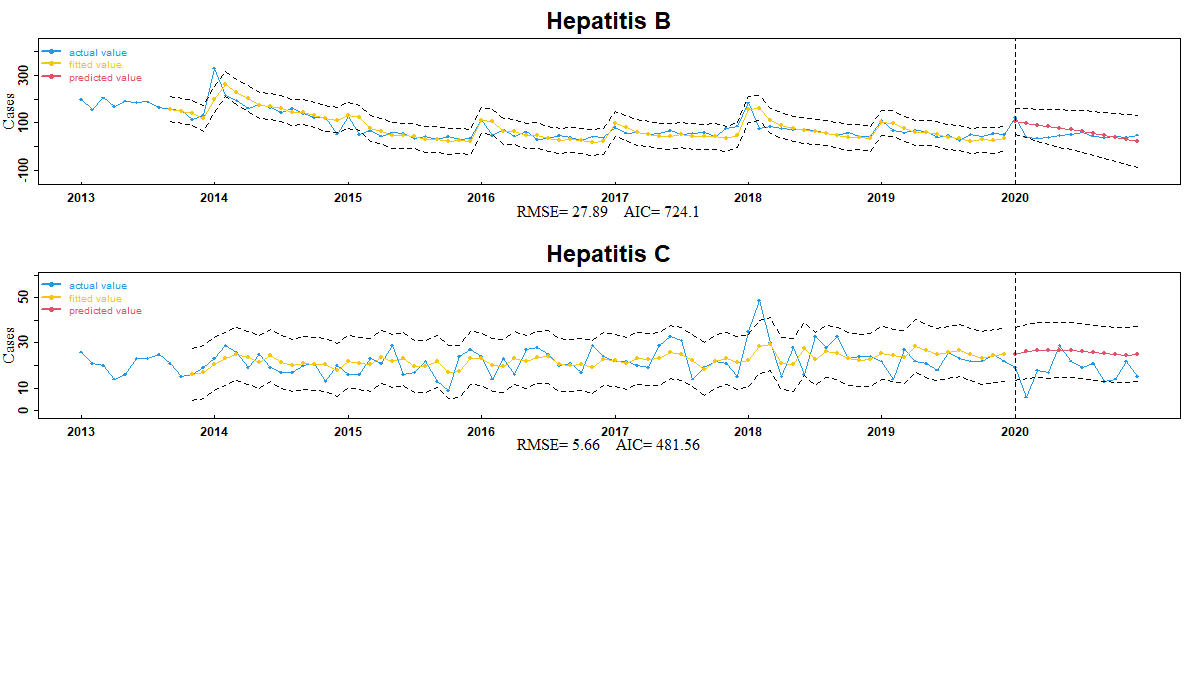
 **Figure S3** The observed and fitted trends of different notifiable infectious diseases from 2013 to 2020 in Yinchuan, China.


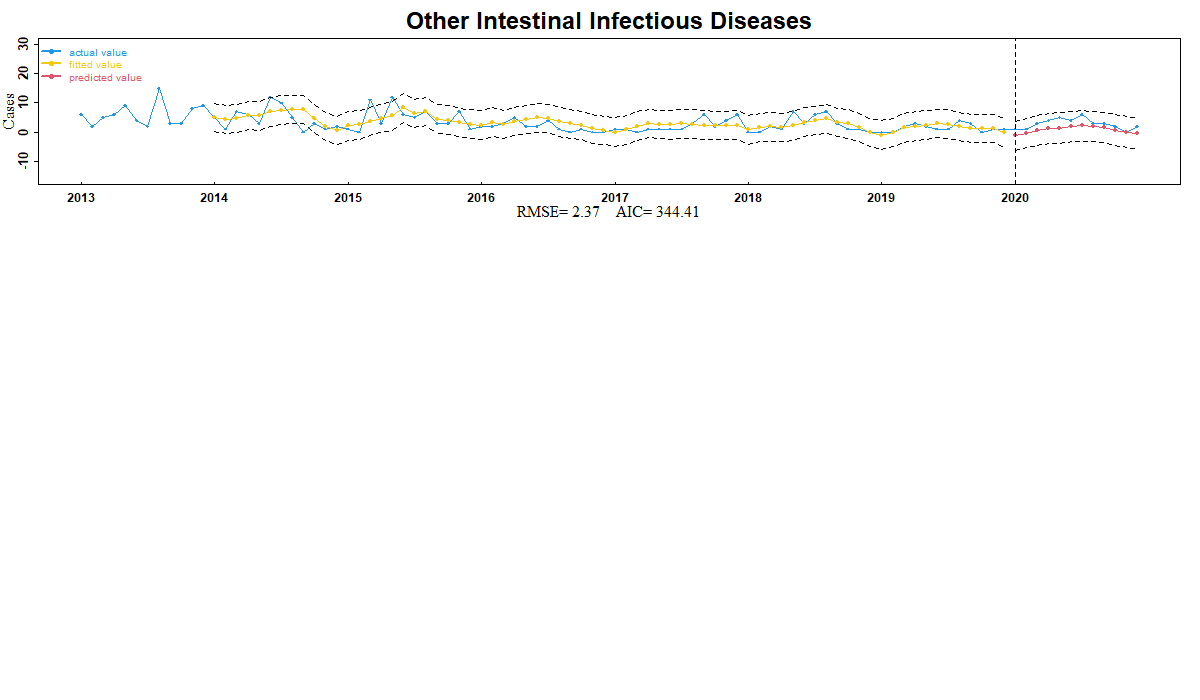


**Respiratory infectious disease**

**Intestinal infectious disease**

**Sexually transmitted or bloodborne diseases**

**Natural focal diseases and vector-borne infectious diseases**

**(a)**

**(b)**

**(c)**

**(d)**


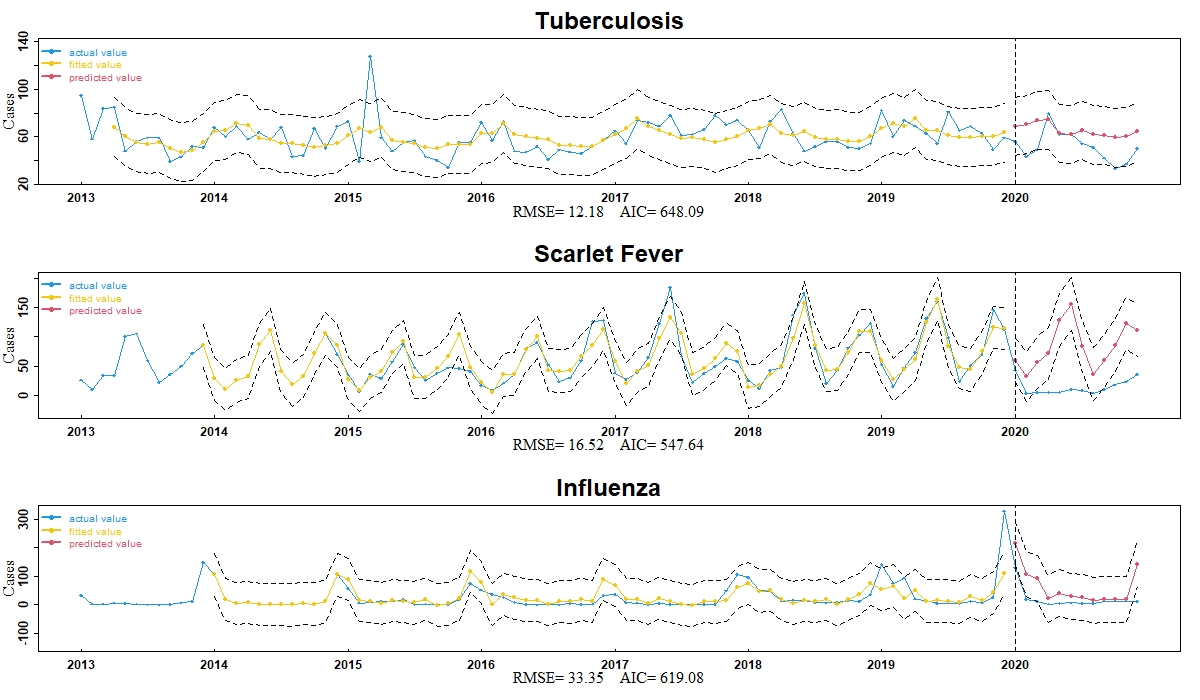

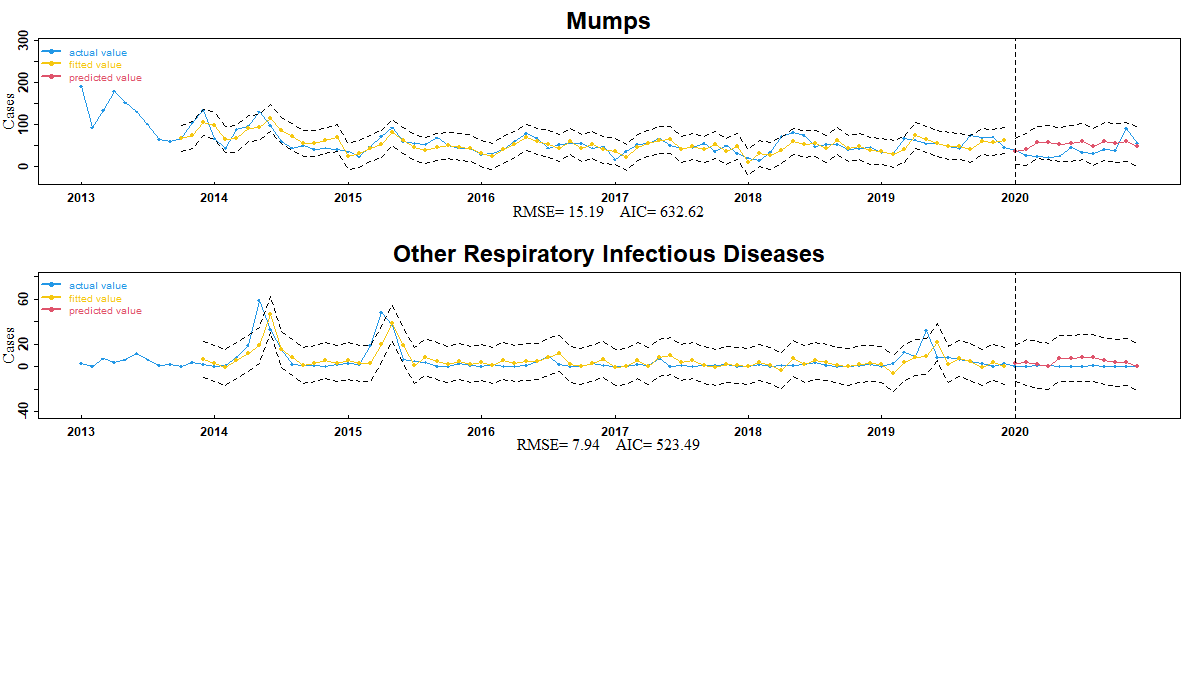

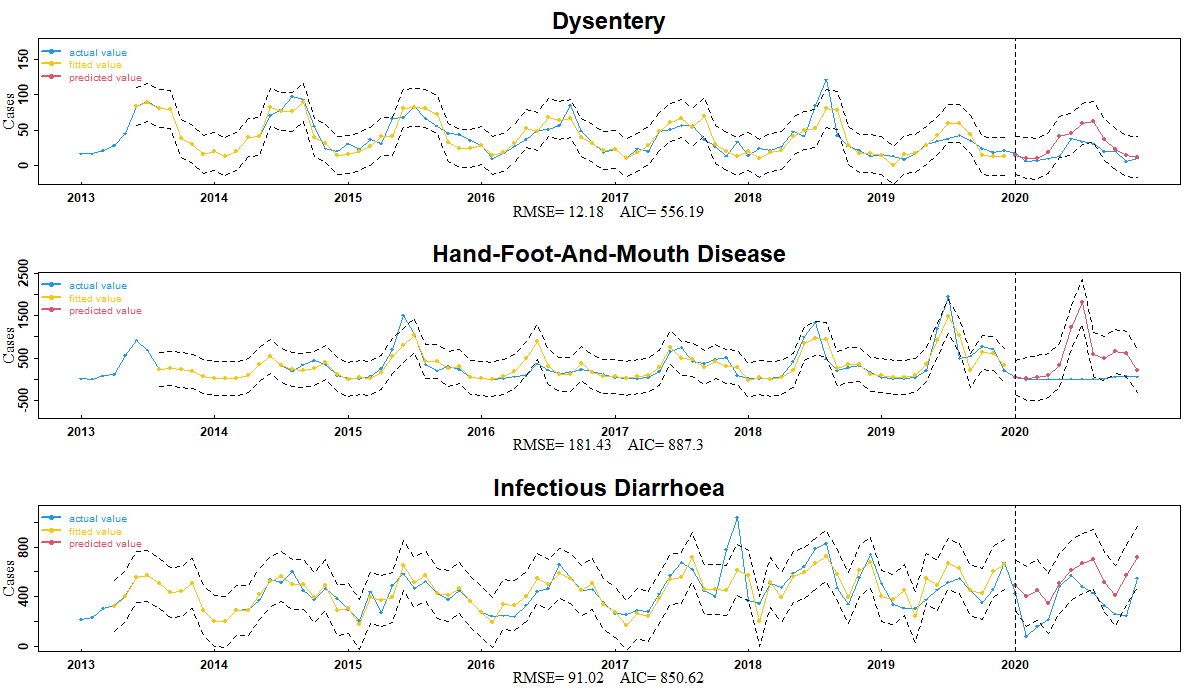

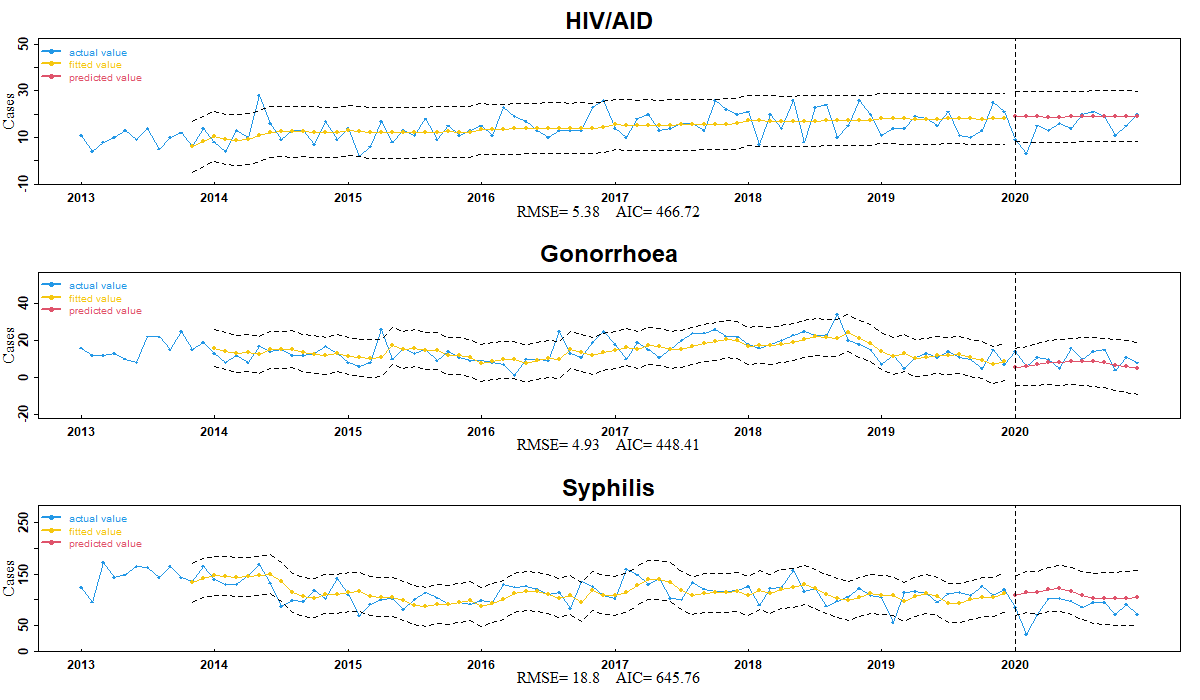

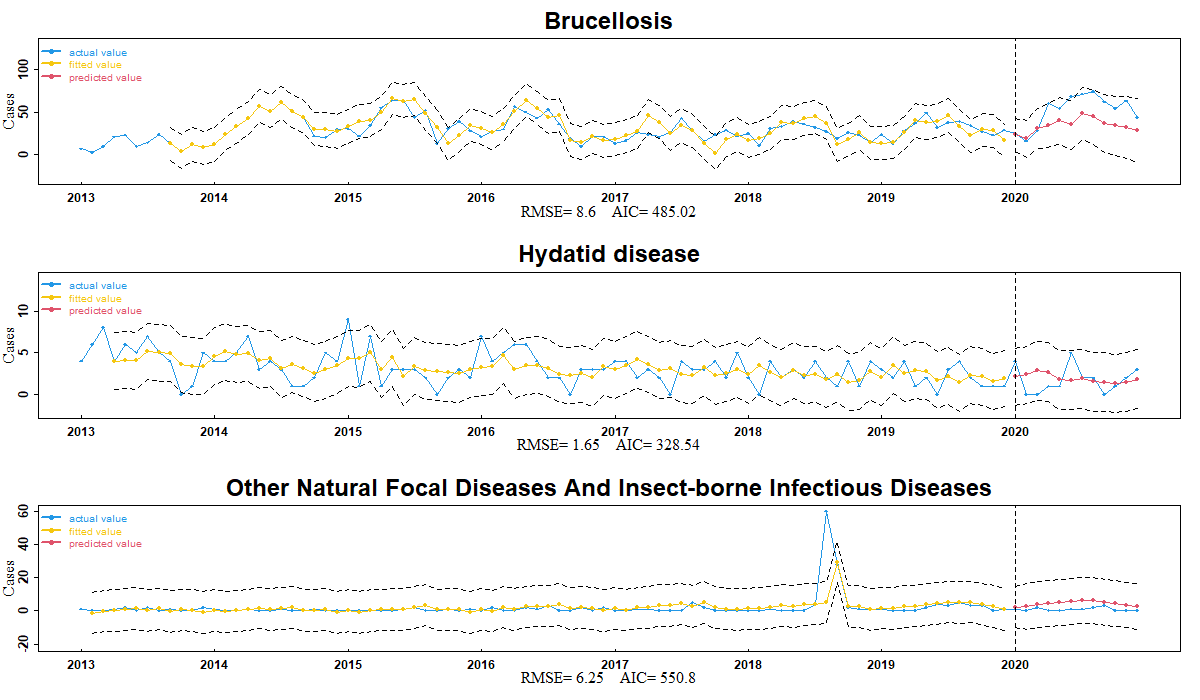


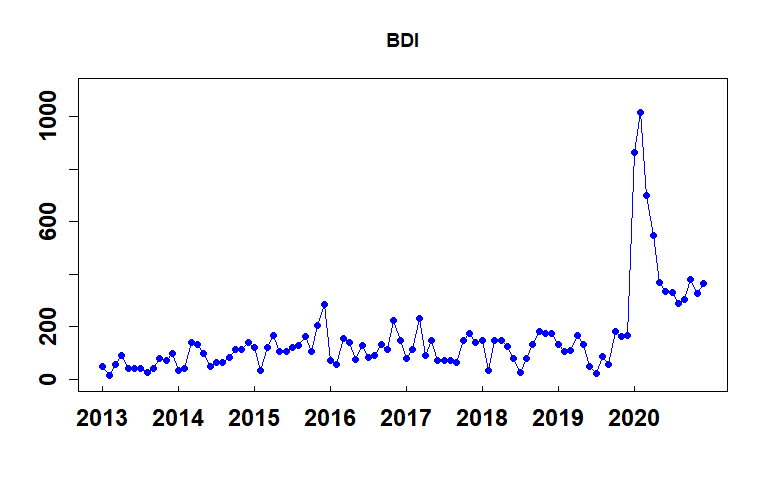


**Figure S4** The time series of monthly Baidu index in Yinchuan of “mask” from 2013 to 2020.

**A1. Baidu index**

In recent years, with the rapid development of the Internet and increasing public health awareness, many people have started to obtain health information through Internet search, which greatly reflects the searcher's physical condition, health awareness and other information, and have been widely applied in epidemiological research^[1-3]^. Baidu is the most popular search engine in China, covering more than 90% of Internet users, whose data can provide timely and effective information delivery channels for epidemic monitoring, emergency response, and public opinion guidance. Additionally, previous studies showed that the incorporation of Baidu index^[4]^ may significantly improve the accuracy of prediction models associated with coronavirus epidemic^[3]^. As a result, we further added the daily Baidu index by selecting "mask" as a keyword in this study, and compared the model including Baidu index with the baseline model to measure the impact of public awareness on the onset of NIDs. The time series of monthly Baidu index in Yinchuan of “mask” from 2013 to 2020 was presented in Figure S4.

1. Zhou W, Zhong L, et al. **Early warning and monitoring of COVID-19 using the Baidu Search Index in China**. J Infect 2022; 84(5):e82-e84.

2. Tu B, Wei L, et al. **Using Baidu search values to monitor and predict the confirmed cases of COVID-19 in China: evidence from Baidu index**. BMC Infect Dis 2021; 21(1):98.

3. Fang J, Zhang X, et al. **Baidu Index and COVID-19 Epidemic Forecast: Evidence From China**. Front Public Health 2021; 9:685141.

4. Baidu. **Baidu Index.** <https://index.baidu.com/>. Accessed 11 Apr 2023. .
